# Relationship between socioeconomic, demographic, health and social characteristics and ability to access reliable information on herbal and food supplements: analysis of Thai Health Literacy Survey 2019

**DOI:** 10.1186/s12889-022-13454-9

**Published:** 2022-05-26

**Authors:** Roongnapa Khampang, Saichon Kloyiam, Rukmanee Butchon

**Affiliations:** 1 Health Intervention and Technology Assessment Program, Ministry of Public Health, Nonthaburi, Thailand; 2 Department of Health, Ministry of Public Health, Office of DoH 4.0 and Health Literacy, Nonthaburi, Thailand

**Keywords:** Health literacy, Accessing information, Herbal, Food supplement, Survey, Thailand

## Abstract

**Background:**

The consumption of herbal and food supplements attributing to health expenditures in Thailand has been increasing over the years. However, information on herbal and food supplement products can make it difficult for some people with limited health literacy to use. Evidence from previous studies outside Thailand shows that SES disadvantaged groups are more likely to have limited health literacy compared with their counterparts with advantaged SES. The present study adds to this body of knowledge through an exploration of health literacy competencies related to herbal and food supplement consumption to determine what competency would be most problematic among Thai people. The study also investigated the influences of demographic and socio-economic factors on the most problematic health literacy competency on herbal and food supplements.

**Methods:**

The THL-S used a stratified three-stage-sampling to draw a sample of Thais aged 15 years and above. Participants were interviewed with a questionnaire of 34 items measuring health literacy and 8 items measuring behavioural practices. Responses to questions on accessing, understanding, communicating, and making decisions related to herbal and food supplement consumption were analysed. A logistic regression model was used to explore the association between having difficulties in accessing information and participant’s socio-economic factors.

**Results:**

Levels of difficulties the participants experienced varied among their health literacy competencies. Accessing reliable information was found to be the most problematic health literacy competency faced by respondents (48%), followed by asking healthcare providers about herbal products and food supplements (41%). Significant differences in the ability to access reliable information on herbal and food supplements were found to be based on differences in: education, income levels, occupation, insurance scheme coverage, age, sex, reading ability, writing ability, chronic diseases, wearing eyeglasses or lenses, hearing impairment, and having a community leading role.

**Conclusions:**

Accessing reliable information on herbal and food supplements has been found to be the most difficult health literacy competency among respondents to the survey, particularly vulnerable consumers in the society such as people with hearing impairment or having limited overall literacy competencies. Therefore, health literacy programs might be developed to build health literacy competencies and empower vulnerable consumers for reasonable use of herbal and food supplements.

## Background

The consumption of food and herbal supplements has been increasing in many countries and becoming a global concern [1]. Although some supplements have acceptable safety profiles, many have been reported to adversely interact with prescribed drugs [2–5]. Their potential benefits are publicly promoted, but their possible harmful effects are less shown. Over-the-counter food and herbal supplements may increase health risks if they are consumed more than the recommended dietary amount [6, 7].

In Thailand, there have been efforts to increase awareness and knowledge on herbal and food supplements to empower people for their reasonable use. However, according to the Thai National Health Examination Survey 2014, the percentage of Thais aged 15 years and above who consumed herbal and food supplements within 30 days increased by about two-fold over a 5 year timescale (14.8% in 2010 versus 33.3% in 2014) [8, 9]. Moreover, out-of-pocket expenses of Bangkok households on consumption of such supplements accounted for one-third of the total amount of out-of-pocket expenses on health care [10]. In addition, a 2017 survey on health behaviour among Thais aged 15 years and above across Thailand revealed that among the 512 interviewees, 70% believed that food supplements were necessary. This belief was found to be most prevalent among those aged 21–30 years (74%), followed by those aged 31–40 years (72%). It was also found that more respondents with a bachelor’s degree or higher possessed this belief compared with those with lower education levels [11].

In a highly competitive market of herbal and food supplements, individuals and companies use marketing strategies, social media, and easy-to-understand formats to draw customer’s attention. There is plenty of information on herbal and food supplements but the trustworthiness of such information has been found to be questionable [12]. Some people pay attention to pictures, colours, and celebrities on product labels and advertisements. This makes it difficult to distinguish reliable information and access reliable sources, especially for those with limited health literacy skills. It also leads to over-consumption of food supplements and herbal medicine. Therefore, there is a need to ensure reliable, accessible, and understandable health information on herbal and food supplements and strategies to empower consumers at high risk.

Previous studies suggested that people were not aware about the negative effects related to herbal products and dietary supplements and initiated consuming these products based on recommendations of friends or relatives rather than asking health care professionals [13, 14]. Also, study participants did not consider the importance of informing their physician or pharmacist about the use of herbal remedies and dietary supplements [14]. These findings highlight the importance of understanding health literacy competencies of Thai people. Health literacy has been defined as “people’s knowledge, motivation and competencies to access, understand, appraise and apply health information in order to make judgments and take decisions in everyday life concerning healthcare, disease prevention and health promotion to maintain or improve quality of life during the life course” [12]. Existing literature shows that socioeconomic status (SES) might contribute to health disparities among population groups [13–15]. However, the manner in which SES affects health status seems to be mediated by other factors [16]. Health literacy has been found to be one of the potential mediators between SES and overall health [16–19]. Evidence from previous studies shows that disadvantaged SES groups are more likely to have limited health literacy compared with their higher SES counterparts [19, 20]. Furthermore, limited health literacy has been reported to be associated with: unhealthy lifestyles; more use of emergency care; lower use of preventive healthcare (mammography screening and influenza vaccine); poorer management of chronic diseases; and higher hospitalization rates and mortality [21–25]. However, little is known about how health literacy affects decision making among population groups when herbal and food supplements are concerned. While herbal and food supplements are easily accessible and have been contributing to health expenditure in countries such as Thailand, those with lower SES seem to be most targeted. Some studies found that health literacy correlated with desired skills such as: estimation of portion size, access to reliable information about food products, understanding food labels, and seeking/trusting nutrition sources [15–18].

A study from Thailand found that the ability to access and appraise health information was associated with reasonable use of food supplements [18]. In addition, socioeconomically disadvantaged groups were less likely to have skills to access and appraise health information. Therefore, the study suggested that improving knowledge and skills to access and appraise health information among socioeconomically disadvantaged groups might be needed.

However, to the best of our knowledge after conducting a literature review, the number of people facing difficulties in accessing, understanding, communicating, and deciding about herbal and food supplements is unclear among the general Thai population. In addition, little is known about how and to what extent health literacy competencies relate to: socioeconomic, demographic, health, and social characteristics of consumers. Therefore, understanding these relationships would not only help us to better understand health literacy but enable us to effectively design, plan, implement, and evaluate programs and activities that empower Thai consumers to smartly use herbal and food supplement products for their optimal health and that of others.

This study explored health literacy competencies related to herbal and food supplement consumption to find out what health literacy competency is problematic among Thai people. We then investigated the relationships between health literacy competency with regards to herbal and food supplement consumption in relation to socioeconomic, demographic, health, and social factors, using data from the 2019 Thai Health Literacy Survey among Thais aged 15 years and above .

## Methods

### Study design and data collection

The Thai Health Literacy Survey used a stratified three-stage sampling model to draw a sample of Thais aged 15 years and above [19]. The study participants have been drawn from all 13 health regions, as geographically categorised by the Ministry of Public Health. In the first stage, three provinces in each health region were systematically identified by ranking the number of inhabitants aged 15 years and above from smallest to largest. Thirty-seven provinces were selected in total. In the second stage, 492 enumeration areas within the selected provinces were defined based on their locations. In the third stage, a total of 7380 households were systematically selected from the 492 enumeration areas. In each household, all members who were 15 years old or older were interviewed using a questionnaire. Data were collected through face-to-face interviews at the participant’s home between March to August 2019. A total of 7295 households participated in the survey of which a total of 18,832 people met the inclusion criteria. The inclusion criteria were that the participants were 15 years old and above, possessed Thai nationality, and had been living in their current addresses for the last 6 months at the interview date. The enumerators were able to interview 17,530 people (response rate of 93%). Of the non-respondents, 867 people were unavailable after a three-time follow-up and 375 people refused to participate in the interview.

### Assessment of variables

#### Health literacy

The questionnaire contained 42 items of which 34 items measured health literacy competencies in four domains, while 8 items measured behavioural practices. The questionnaire was developed based on the Health Literacy Conceptual Framework proposed by the Department of Health, Ministry of Public Health that was adapted from the integrated model of health literacy proposed by Sorensen, 2012 [20]. The content of the questionnaire had been discussed and revised based on consultations from four expert panels. Each panel included four health professionals from the Department of Health, four health literacy experts from Mahidol University, an international health literacy expert from Deakin University, and two health system and policy researchers from the Health Systems Research Institute (HSRI) and the Heath Intervention and Technology Assessment Program (HITAP). The items measuring health literacy competencies in the herbal and food supplement consumption are shown in Table 1. The questionnaire was pre-tested for understandability and relevance with a sample of 722 people from six provinces across all regions (*n* = 120 in each province). Focus groups were conducted with 10–12 respondents who had difficulty understanding the questionnaires. The overall internal reliability of the questionnaire, as indicated by Cronbach’s alpha coefficient of 0.94, was found to be satisfactory. The internal reliability for the health literacy competencies was also good (Cronbach’s alpha coefficient of 0.88 for ‘accessing’, 0.86 for ‘understanding’, 0.90 for ‘communicating’ and 0.88 for ‘making health-related decisions’). For accessing, understanding, and making health-related decisions, the respondents were asked to choose from a 6-point Likert scale ranging from,1 = very easy, 2 = fairly easy, 3 = fairly difficult, 4 = very difficult 5 = unable to perform, and 6 = confident in performing but never had a chance to perform. For communicating, the 6-point Likert scale ranged from, 1 = all the time, 2 = sometimes, 3 = never do, 4 = do not dare to do, 5 = don’t want to do, and 6 = having someone do it.

**Table 1:** Health literacy competency domains related to consumer protection

Competency domain	On a 6-Likert scale from very easy to very difficult,
	1. = very easy
	2. = fairly easy
	3. = fairly difficult
	4. = very difficult
	5. = unable to perform
	6. = confident in performing but never had a chance to perform how easy is it for you to …
Accessing	Find reliable information about medicine, cosmetic products, herbal products and food supplements.
Understanding	Understand information on labels of medicine products, cosmetic products, herbal products, medical devices, and hazardous chemical products.
Communicating	Inquire healthcare providers about health-related products
Making decisions	Make decisions about what food supplements or herbal products are suitable for you.

#### Demographic and socio-economic characteristics

The demographic and socio-economic characteristics analysed in this study were: sex, age group, marital status, the highest level of education, ability to read and write, holding a leadership role in the community, income sufficiency, occupation, insurance scheme, chronic diseases, having hearing impairment, and use of corrective eyewear. All of the demographic and socio-economic data were analysed as categorical variables.

### Statistical analysis

Proportions of responses (the 6-Likert scales) were calculated for all competency domains. A logistic regression model was performed to explore the association between having difficulties in accessing reliable information and socioeconomic, demographic, health and social characteristics because accessing information has been found to be the most difficult health literacy competency among respondents to the survey. The responses to each question were grouped into a dichotomous variable representing whether respondents had difficulties in accessing information. The response 1 and 2 (very easy and fairly easy) were grouped into one category to signify not having difficulty, response 3–5 (fairly difficult, very difficult and unable to perform) were grouped to signify having difficulty. In addition, response 6 (confident in performing but never had a chance to perform) was treated as not applicable because the respondents had not actually demonstrated their competencies in performing the mentioned task. A binomial logistic regression was performed using R 3.1.0 [21, 22]. The primary exposures were age group, occupation, the highest level of education, and ability to read and write. Potential confounders were sex, marital status, income sufficiency, insurance scheme, chronic diseases, use of corrective eyewear, having hearing impairment and holding a leadership role in the community.

## Results

The demographic and socio-economic characteristics of the sample are shown in Table 2. Of the study participants, 61% were female. This proportion was larger than that of overall Thai population, where females constitute 51% of the total population [23]. In terms of age groups, most participants were 60 years old and above, followed by those aged between 46 and 59 years. These figures were not in line with the distribution of the Thai population: the proportion of the study participants aged 60 years and above and aged 46–59 years was larger while that of those aged 25–45 years and 15–24 years was smaller. The majority of the study participants were married or were living together. Half of the participants had completed primary education while 4% were illiterate. Approximately 10% of the study participants reported insufficient income to support their family and a third of the participants worked in the agricultural sector. The majority of the participants (79%) were registered under the Universal Coverage Scheme (Thai social security health scheme).

**Table 2:** Characteristics of study participants

Characteristics	Total (***n*** = 17,530)		Male (***n*** = 6779)		Female (***n*** = 10,751)	
	%	n	%	n	%	n
**Age group**						
15–24 years	10.9	1906	12.3	832	10.0	1074
25–45 years	24.0	4208	25.2	1707	23.3	2501
46–59 years	30.2	5302	27.8	1887	31.8	3415
60 years and above	34.9	6114	34.7	2353	35.0	3761
Missing values	0	0	0	0	0	0
**Marital status**						
Single	21.5	3764	24.8	1678	19.4	2086
Married or living together	63.3	11,099	67.7	4588	60.6	6511
Separated or divorced	15.1	2651	7.5	507	19.9	2144
Missing values	0.1	16	0.1	6	0.1	10
**Highest level of education**						
No education	4.0	702	2.8	190	4.8	512
Primary education	51.6	9043	47.8	3240	54.0	5803
Lower secondary education	13.0	2276	15.8	1073	11.2	1203
Upper secondary education	19.1	3347	21.2	1436	17.8	1911
Tertiary education	12.3	2150	12.3	835	12.2	1315
Missing values	0.1	12	0.1	5	0.1	7
**Income sufficiency**						
Deprived	11.7	2051	10.4	706	12.5	1345
Sometimes sufficient	47.0	8241	47.4	3212	46.8	5029
Often sufficient	35.1	6157	36.2	2454	34.4	3703
Saving	6.0	1056	5.9	398	6.1	658
Missing values	0.1	25	0.1	9	0.1	16
**Occupation**						
Unemployed	20.0	3503	12.6	854	24.6	2649
Agriculture	33.7	5899	35.6	2410	32.5	3489
Business owner	12.9	2253	10.1	682	14.6	1571
Casual employment	18.6	3257	25.0	1693	14.5	1564
Government officer	4.7	820	5.6	378	4.1	442
Employees of private companies	3.7	654	4.5	303	3.3	351
Student	6.3	1098	6.4	435	6.2	663
Others	0.2	35	0.3	18	0.2	17
Missing values	0.1	11	0.1	6	0.0	5
**Insurance scheme**						
UCS^a^	79.0	13,842	78.1	5293	79.5	8549
SSS^b^	9.8	1716	10.4	706	9.4	1010
CSMBS^c^/ State enterprise	8.6	1516	8.8	594	8.6	922
Private insurance and others	2.6	456	2.7	186	2.5	270
Missing values	0.0	0	0.0	0	0.0	0

^a^*UCS* Universal Coverage Scheme; ^b^*SSS* Social Security Scheme; ^c^*CSMBS* Civil Servant Medical Benefit Scheme

The summary of responses to questions of the four competencies (‘accessing’, ‘understanding’, ‘communicating’ and ‘making health-related decisions’) related to herbal and food supplement consumption is presented in Table 3. The proportions show that level of difficulties the participants experienced (responses of difficult, very difficult and unable to perform) varied across the health literacy competencies, with accessing information being the most difficult while comprehensions was the least difficult. Almost half the study participants (48%) were unable to, or experienced difficulties in accessing reliable information related to herbal and food supplements. Forty-one percent of the study participants reported having difficulties in asking healthcare providers about herbal products and food supplements.

**Table 3:** Distribution of responses to questions on four competency domains related to consumer protection (Percentage of total responses to each domain)

Competency domain	Items	Very easy	Fairly Easy	Fairly Difficult	Very difficult	Unable to perform	Confident in performing but never had a chance to perform
Accessing	Find reliable information about medicine, cosmetic products, herbal products and food supplements	11.7	33.7	11.0	4.6	32.4	6.6
Understanding	Understand information on labels of medicines, cosmetic products, herbal products, medical devices, and hazardous chemical products	25.3	53.8	11.2	3.6	4.3	1.8
Communicating	Inquire healthcare providers about health-related products	13.8	39.9	37.5	1.9	1.8	5.2
Making decisions	Able to choose suitable food supplements or herbal products	17.8	54.7	12.9	3.2	3.8	7.6

Significant associations with the degree to which individuals accessed reliable information related to herbal and food supplements were found among: education, adequacy of income, occupation, insurance scheme, age, sex, reading ability, writing ability, chronic diseases, visual impairment, hearing impairment, and having community leadership roles as shown in Table 4. People with a lower level of education or inadequate income tended to report greater difficulties in accessing reliable information compared to their counterparts. Similarly, people working in the agricultural sector had a higher chance of reporting difficulties in accessing reliable information than students or other occupations. Governmental officers appeared to experience fewer difficulties in accessing information compared to students or other occupations.

**Table 4:** Associations between socioeconomic, demographic, health and social characteristics and having difficulty in accessing information related to health care products e.g. medicines, herbs, or cosmetics

Factors	Having difficulty in accessing information					
	Crude OR	95% CI		Adjusted OR	95% CI	
		Lower	Upper		Lower	Upper
**Highest completed education (reference group: tertiary education** ***n*** **= 2028)** 51.4% reported having difficulty						
No education (*n* = 666)	29.14*	22.94	37	3.08*	2.24	4.22
Primary education (*n* = 8404)	14.29*	12.53	16.29	3.46*	2.94	4.08
Lower secondary education (*n* = 2057)	3.12*	2.68	3.64	1.93*	1.63	2.3
Upper secondary education (*n* = 3104)	1.88*	1.62	2.17	1.44*	1.22	1.69
**Level of adequacy of income (reference group: saving)**						
Deprived	4.44*	3.76	5.23	1.71*	1.4	2.09
Sometimes sufficient	2.65*	2.3	3.05	1.50*	1.26	1.78
Often sufficient	1.66*	1.43	1.92	1.14	0.96	1.36
**Occupation (reference group: student and others)**						
Agriculture	10.53*	8.8	12.6	1.31*	1.01	1.7
Unemployed	12.74*	10.57	15.34	1.21	0.93	1.57
Business owner	4.12*	3.4	4.99	0.88	0.67	1.15
Casual employment	5.63*	4.68	6.77	1.02	0.79	1.32
Governmental officer	1.33*	1.04	1.71	0.71*	0.51	0.98
Employees of private companies	1.09	0.82	1.44	0.8	0.56	1.13
**Insurance scheme (reference group: private insurance and others)**						
UCS^a^	3.36*	2.7	4.17	1.54*	1.19	2
SSS^b^	0.84	0.66	1.08	1.16	0.87	1.55
CSMBS^c^	2.45*	1.93	3.11	1.41*	1.06	1.89
**Age group (reference group: 15–24 years old)**						
25–45 years	1.87*	1.61	2.16	1.68*	1.37	2.07
46–59 years	5.94*	5.17	6.83	3.48*	2.81	4.31
60 years and above	18.55*	16.1	21.36	6.78*	5.43	8.47
**Male**	0.95	0.89	1.01	1.11*	1.03	1.2
**Marital status (reference group: single)**						
Married or living together	3.2*	2.94	3.48	1.09	0.97	1.23
Separated or divorced	5.83*	5.21	6.53	1.19*	1.02	1.38
**Reading ability (reference: fluent)**						
Cannot read	8.84*	7.38	10.58	2.17*	1.5	3.13
Can read but not fluent	5.98*	5.48	6.51	1.77*	1.48	2.13
**Writing status (reference: fluent)**						
Cannot write	9.1*	7.51	11.03	1.27	0.87	1.85
Can write but not fluent	5.73*	5.29	6.21	1.22*	1.02	1.46
**Presence of chronic diseases (reference group: don’t know because never received screening)**						
No chronic diseases	1.07	0.99	1.16	0.87*	0.78	0.96
Having diagnosed with chronic diseases	2.63*	2.42	2.86	1.01	0.91	1.13
**Use of corrective eyewear**	1.3*	1.22	1.39	0.84*	0.77	0.92
**Having hearing impairment**	3.23*	2.82	3.7	1.35*	1.15	1.58
**Having community leadership roles**	0.67*	0.62	0.73	0.57*	0.52	0.62

* *p*-value < 0.05

^a^*UCS* Universal Coverage Scheme, ^b^*SSS* Social Security Scheme, ^c^*CSMBS* Civil Servant Medical Benefit Scheme

Nagelkerke R Square for accessing model = 0.40

In addition, people under the Civil Servant Medical Benefit Scheme and UCS were more likely to experience difficulties in accessing information compared to those with private insurance schemes. Similarly, people at an older age, who were male, unable to read, or having hearing impairment, were more likely to experience difficulties in accessing reliable information than their counterparts.

In contrast, people who had no chronic diseases, wore corrective eyewear, or had community leadership roles were less likely to report difficulties in accessing such information compared to their counterparts.

## Discussion

The present study identified factors associated with health literacy competencies specifically focusing on accessing reliable information related to herbal and food supplements using data from the 2019 Thai Health Literacy Survey among Thais aged 15 years and above. It addressed the relationship of socioeconomic, demographic, health, and social characteristics on how Thais might access information on herbal and food supplements and how it might be related with the consumption of herbal and food supplements. First, we found that most participants reported difficulties in accessing reliable information on herbal and food supplements, followed by them having difficulties in asking healthcare providers about these products. This indicates that more attention might be paid to making reliable information on herbal and food supplements more accessible and promoting friendly environments to ask questions or have discussions about the benefits and side effects of herbal and food supplements both in the health care system and communities.

Second, the study results showed significant associations of education, adequacy of income, occupation, insurance scheme, age, sex, reading ability, writing ability, chronic diseases, wearing corrective eyewear, hearing impairment, and having a community leadership role on accessing information about the herbal and food supplements. These factors can be considered for developing health literacy-friendly strategies for the reasonable use of herbal and food supplements.

Similar relationships between the health literacy competency and education, age group, reading ability, writing ability, hearing impairment, visual impairment, and having a community leadership role were found in previous studies in other countries. The explanations for these relationships have been studied elsewhere [24–28].

Reading and writing ability is seen as a part of functional health literacy. The focus is on the ability to read basic texts and write simple statements on everyday life. Those who read and write well are able to find and use available text-based health information and services [29].

The present study found that those with higher educational levels seemed to have better access to reliable information on herbal and food supplements. However, a previous study found that those with a bachelor degree or higher were more likely to report that herbal and food supplements were necessary compared with those with lower education levels [20].

Another interesting finding was that the participants aged 60 years and above had more limited access to reliable information compared to participants aged 15–24 years old. The elderly might not be competent using the Internet. Most resources of health information are online. They might also lack skills to evaluate the reliability of the information they received. Factors such as marketing strategies (price, quality, quantity) of herbal and food supplements might have explained this finding as found by previous studies [30, 31].

Regarding occupation, only those in the agricultural sector were found to be more likely to report difficulties accessing reliable information on herbal and food supplements while governmental officers were less likely to have difficulties in accessing information compared to students. This might have to do with the nature of work of these occupations and how marketing strategies of herbal and food supplements are shaped around these groups. Individuals need essential knowledge and skills to be able to seek, find, and obtain reliable health information such as: reading, writing, numeracy, and communication skills. These skills can be built and practiced daily [32]. Governmental officers who work with information and practice such skills more often would have developed a higher level of those skills. This might explain why fewer governmental officers reported having difficulties accessing information compared to students and those in the agricultural sector.

The present study found that male participants were more likely to have more difficulties in accessing reliable information than female participants, while another study found no significant difference between males and females after controlling for education and existence of chronic illness among respondents [33]. The association between sex and health literacy competencies related to herbal and food supplements should be explored further with qualitative studies to help develop sex - specific strategies for reasonable use of herbal and food supplements.

Interestingly, the present study found that those without chronic diseases (confirmed by health screenings) were less likely to report difficulties in accessing information related to herbal and food supplement products than people who had never received a health screening. This is consistent with the findings of the Thai National Health Literacy Survey [19]. A possible explanation for this relationship is that people who are aware and concerned about their health have explored more information sources. However, some previous study found that people who reported one or more chronic conditions had a better knowledge about their chronic diseases [34] and another study explained that having chronic diseases motivated patients to learn and engage more with self-management [20].

Individuals with disabilities already face issues regarding access and poorer outcomes because of their disabilities [35, 36]. This is in line with our finding that people having hearing impairment experienced more difficulties in accessing information. Those with visual impairment, if not assisted with corrective eyewear, will also face barriers in accessing health information.

The present study recommends that skills such as searching for reliable sources and assessing the reliability of information on herbal and food supplements as well as asking for clarification from healthcare providers might be needed and should be explored in future research. Additionally, a study on the effectiveness of establishing friendly environments with a shame-free atmosphere where people can ask questions might be useful in Thai health care settings. A good example is the ‘Ask Me Three’ approach, a practice that encourages patients and family members to ask three specific questions to better understand their health conditions. The practice has been found to be effective in improving patient’s understanding, communication skills, and compliance with health-related advice [37]. It is also recommended that health professionals might assess service user’s health literacy before giving advice or information on herbal and food supplements. We cannot assess individual’s health literacy by looking at one’s occupation, income, occupation, or outfit. Everyone benefits from easy-to-understand health information. Therefore, clear communication is recommended. Reliable sources of information are important for consumers to gain knowledge about herbal and food supplement products. The information should be easy to access in communities for those with various health literacy needs. Another recommendation is to promote and build skills and knowledge of the population for evaluating health information on herbal and food supplements. Finally, a monitoring and alert system for consumers about untrustworthy information of herbal and food supplement products on the Internet and communities might be developed. Priority should be given to vulnerable people in the society such as those who work in the agricultural sector, are older, are male, have a low level of education, have insufficient income, have hearing or visual impairment, limitations in reading or writing, and have no engagement in the community.

### Strengths and limitations of the study

This study used the data from the Thai Health Literacy Survey 2019. The large sample size of this survey would ensure the reliability of the data and accuracy of the estimates.

Another strength comes from the questionnaire being administered through face-to-face interviews of participants identified from a three-stage sampling technique based on health regions, provinces, and enumeration areas. This allowed for collecting data from across the country and the face-to-face modality allowed some minorities who might have inadequate reading and writing abilities in Thai language to be able to participate in the study.

A limitation of this study is that the majority of the study participants consisted of the elderly, which might have affected the results of other factors such as adequacy of income, level of education, occupation, and ability to read and write. The questionnaire did not include some variables that might have affected access to reliable information such as experiences of taking care of ill people in the family, duration of living with the disease [38], and media health literacy [37]. It is possible that the study participants responded to the questions in a manner that was considered socially desirable. In addition, as cross – sectional, there is no evidence of the causal relationship between the factors and the outcome, and the study is susceptible to biases such as misclassification due to recall bias.

## Conclusions

Accessing reliable information on herbal and food supplements has been found to be an issue encountered by most respondents. Vulnerable consumers in the society, such as people who work in the agricultural sector, are older, are male, have a low level of education, have insufficient income, have hearing impairment, have limitations in reading or writing, are more likely to face difficulties in accessing reliable information. Health literacy among other social determinants of health could also impact health status among vulnerable populations. Health literacy programs might be developed to build health literacy competencies and empower vulnerable consumers for the reasonable use of herbal and food supplements. Organizations responsible for improving health literacy might also promote adult education among elderly people to improve basic literacy skills, knowledge, social skills, motivation to access reliable information, and health communication skills. Furthermore, there might be a need to adapt the current health services and information on herbal and food supplement products to meet health literacy needs among vulnerable consumers. In addition, reliable health information on herbal and food supplement products can be made more accessible in communities.

## Data Availability

The data analysed for this study are not publicly available due to not obtaining ethical approval to share data publicly but can be available from the corresponding author on reasonable request. The survey was carried out under the jurisdiction of coauthors. The survey questionnaire was developed by the Faculty of Public Health, Mahidol University in collaboration with Department of Health, Ministry of Public Health. The questionnaire is only available in Thai at https://www.hsri.or.th/researcher/research/new-release/detail/11454 .

## Abbreviations

THL-S: Thai Health Literacy Survey
HLS-EU: European Health Literacy Survey
UCS: Universal health care coverage scheme
SSS: Social Security Scheme
CSMBS: Civil Servant Medical Benefit Scheme
OR: Odds ratio
95% CI: 95% confidence interval
